# Association between low-carbohydrate diet and chronic kidney disease in population with gestational diabetes mellitus history: based on the National Health and Nutrition Examination Survey database

**DOI:** 10.29219/fnr.v69.10986

**Published:** 2025-07-24

**Authors:** Junli Zhang, Youlian Dong

**Affiliations:** 1 Department of Obstetrics, Guangyuan Central Hospital, Sichuan Province, China; 2 Department of Obstetrics, the First People’s Hospital of Xiaoshan District, Hangzhou, China

**Keywords:** chronic kidney disease, cross-sectional study, gestational diabetes mellitus, low-carbohydrate diet, National Health and Nutrition Examination Survey

## Abstract

**Background:**

Gestational diabetes mellitus (GDM) is one of the most common metabolic complications during pregnancy, and is associated with a significantly increased risk of postpartum chronic kidney disease (CKD). Although a low-carbohydrate diet (LCD) is recommended for glycaemic management in GDM patients, its long-term impact on kidney health remains unclear.

**Objective:**

To address this knowledge gap, this study aimed to investigate the association between LCD and the risk of CKD in women with a history of GDM, providing evidence for optimising postpartum dietary intervention strategies.

**Method:**

GDM data from National Health and Nutrition Examination Survey database (2009–2018) were used, with LCD as an independent variable and CKD as a dependent variable. Univariate and multivariate logistic regression analyses were applied to investigate factors related to CKD. Stratified and sub-group analyses were conducted to investigate association of LCD with CDK. Restricted cubic splines (RCS) were utilised to analyse non-linear relationship between the two variables.

**Results:**

There were 701 samples in all (CKD: *n* = 130; non-CKD: *n* = 571). The LCD score and CKD risk were shown to have a significant positive association (Odds Ratio [OR] > 1, *P* < 0.05) in multivariate weighted logistic regression model. The link between LCD score and CKD was strongly impacted by race, body mass index (BMI), and smoking status (*P* for interaction < 0.05). In patients who presently smoke and use alcohol, stratified analysis showed a substantial positive correlation (*P* < 0.05) between LCD score and CKD risk. RCSs curve indicated a potential linear relationship (*P*-non-linear = 0.9561) between risk of LCD and CKD, with an LCD score of 10 serving as the criterion threshold for CKD risk and OR < 1 when LCD < 10, signifying a protective factor.

**Conclusion:**

Among women with GDM, higher LCD scores showed a significant positive correlation with CKD risk, particularly in sub-groups with smoking or alcohol consumption habits. The study suggests the need for careful evaluation of the long-term renal safety of LCD and highlights the importance of developing individualised dietary plans for high-risk populations.

## Popular scientific summary

This study utilised data from the NHANES database (2009–2018) to investigate the association between low-carbohydrate diet (LCD) scores and the risk of chronic kidney disease (CKD) in women with a history of gestational diabetes mellitus (GDM). Key findings include:

1. Positive association: Higher LCD scores were significantly associated with an increased risk of CKD.
2. Modifying factors: Smoking and alcohol consumption amplified the association between LCD and CKD, with stronger correlations observed in these sub-groups.
3. Threshold effect: An LCD score >10 emerged as a critical risk threshold for CKD, with a linear dose-response relationship between the two.
4. Clinical implications: The findings suggest that while LCD is commonly used in GDM management, its implementation should be carefully adjusted to mitigate long-term renal risks, particularly in individuals with unhealthy lifestyle habits.

This study highlights the need for personalised dietary recommendations for the GDM population to balance metabolic benefits with kidney health.

Gestational diabetes mellitus (GDM), a prevalent form of diabetes, is a unique kind of the disease that only develops during pregnancy. It is one of the most frequent metabolic problems during pregnancy and is described as the initial identification of glucose intolerance (1). For women with GDM, food control is now the primary line of therapy (2, 3). According to several national and international standards, in order to prevent their diabetes from getting worse during pregnancy, women with GDM should limit their carbohydrate consumption to 35–45% of total calories, or follow a low-carbohydrate diet (LCD) (4, 5). One of the most prevalent illnesses impacting human health is chronic kidney disease (CKD), a clinical condition characterised mostly by primary or secondary kidney damage that manifests as a progressive, gradual, and irreversible decrease of kidney function (6). One of the most prevalent causes of the rising CKD burden is diabetes, which affected around 130 million CKD patients in 2019 and contributed to over 400,000 fatalities as well as a significant financial and medical burden worldwide (7). Moreover, diabetic nephropathy, one of the primary causes of end-stage renal disease, is thought to be the most frequent complication endangering the lives of patients. It is strongly linked to a higher overall mortality rate as well as the risk of developing and dying from cardiovascular disease (CVD) and its consequences (7–9).

Previous studies have shown a close relationship between the development of CKD and metabolic disorders (10). Since metabolic health is related to diet (11), eating a balanced diet is crucial to improving metabolic syndrome markers and lowering the risk of CKD. The LCD score is significantly positively connected with total mortality in persons with CKD, but not in those without CKD, according to a prior study on the relationship between LCD score and mortality rates in populations with and without CKD (12). Nevertheless, it is still unclear if controlling LCD might affect the chance of developing CKD by altering the dietary pattern that is frequently used to treat GDM. Therefore, to provide fresh insight into dietary management and CKD prevention in this group, this study set out to investigate the association between LCD score and the risk of CKD incidence in people with a history of GDM. This is essential for creating dietary advice and preventative techniques that are specifically tailored for these individuals.

## Methods

### Data source and study population

In order to evaluate the physical and nutritional health of adults and children in the United States, the National Center for Health Statistics (NCHS) of the Centers for Disease Control and Prevention (CDC) conducts the National Health and Nutrition Examination Study (NHANES), an ongoing, nationally representative, cross-sectional survey of around 5,000 individuals every year and every 2 years. All survey respondents gave written, informed consent, and the NCHS Research Ethics Review Board approved the survey. Detailed statistical data can be accessed at http://www.cdc.gov/nchs/nhanes.htm.

**Fig. 1:**
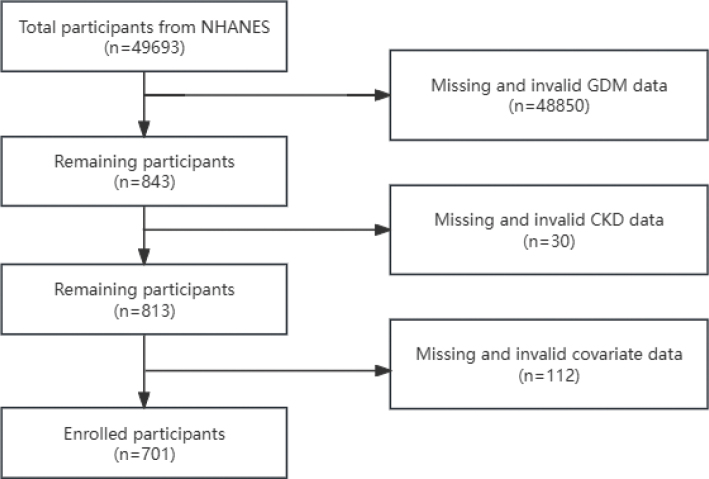
The flowchart of participants.

Data from five NHANES cycles, spanning from 2009 to 2018, were investigated. Out of the 49,693 Americans that took part during this time, only 843 had a verified history of GDM. A total of 701 people entered the study after excluding those with invalid or missing CKD data (*n* = 30) and other participants with missing covariate data, as indicated in Fig. 1 for the particular screening procedure.

### Gestational diabetes mellitus

Responses to the question, ‘During pregnancy, were you ever told by a doctor or other health professional that you had diabetes, sugar diabetes or gestational diabetes?’ with a ‘yes’ were classified as having GDM (13).

### Low-carbohydrate diet

Data from two 24-h food recall interviews were used to compute the average dietary consumption of fat, protein, carbs, and energy (14). The percentages of fat (kcal), protein (kcal), carbs (kcal), and energy were then computed based on the conversion of the intake of fat, protein, and carbohydrates per gram to kilocalories at a ratio of 1:9, 1:4, and 1:4, respectively. For detailed scoring criteria, see Table S1. The LCD score, which runs from 0 to 30, is the total of the three nutritional scores. A higher score corresponds to consumption of more protein and fat and less carbs (15).

### Chronic kidney disease

CKD is characterised by a urine albumin-to-creatinine ratio of less than 30 mg/g or an estimated glomerular filtration rate (eGFR) of 2 (16). The following Chronic Kidney Disease Epidemiology Collaboration (CKD-EPI) equation (17) serves as the basis for eGFR computation:

eGFR = 141 × min(Scr/κ,1)α × max(Scr/κ,1)−1.209 × 0.993Age × 1.018[if female] × 1.159[if black].

The creatinine concentration is represented by Scr. The κ values are taken according to gender, 0.9 for males and 0.7 for females, and α values are taken according to gender, −0.411 for males and −0.329 for females.

### Covariates

Covariates included age (≥20 years), race (Mexican American, other Hispanic, non-Hispanic White, non-Hispanic Black, other race), body mass index (BMI) (Normal: <25kg/m^2^, overweight: 25 to <30kg/m^2^, obese: ≥30kg/m^2^) (18), alcohol drinking (yes, no), smoking (never, former, now) (19), poverty-to-income ratio (PIR) (≤1.3, 1.3–3.5, >3.5) (20).

The term hypertension (21) refers to a condition in which any of the following occurs: 1) self-reporting the use of antihypertensive drugs; 2) having a history of hypertension; and 3) having an average systolic blood pressure of at least 130 mmHg or a diastolic blood pressure of at least 80 mmHg during the NHANES assessment (22).

The following query characterises a family history of diabetes: ‘Including living and deceased, were any of your close biological that is, blood relatives including father, mother, sisters or brothers, ever told by a health professional that they had diabetes?’ Participants are classified as having a family history of diabetes (23) if they select ‘yes’ in response to this question.

Heart attacks, strokes, congestive heart failure (CHF), angina, and coronary heart disease (24) are among the CVD events that participants are asked about. A history of any of these conditions is defined as having a history of CVD.

### Statistical analyses

In this study, baseline tables were created using the ‘tableone’ package, which allowed individuals to be categorised as either having CKD or not, based on population-wide factors. Sample size and percentage (*n* [%]) are used to represent categorical variables, whereas mean and standard deviation (sd) are used to represent continuous variables. Weighted logistic regression models of the association between LCD and CKD in people with a history of GDM were constructed using the ‘survey’ package, with stratified analyses of categorical variables in models that did not adjust for confounders. The interaction term *P*-values of the stratified logistics regression model with all confounders adjusted were subjected to a likelihood ratio test. When *P* < 0.05, a significant difference is seen. The LCD was stratified using weighted tertiles to construct a weighted logistics regression model adjusting for confounding factors for both LCD and CKD, and sub-group analysis of confounding factors was performed. Association between LCD and CKD was explored using RCS. The models in this study included Crude without adjustment; model I adjusted for age, race, BMI, smoking, alcohol drinking, PIR; and model II adjusted for age, race, BMI, smoking, alcohol drinking, PIR, hypertension, CVD events, family history of diabetes. All statistical analyses were performed using R (V4.3.3).

## Results

### Baseline characteristics of participants

This study included 701 women aged 20 years and older with a history of GDM from NHANES database between 2009 and 2018, as shown in Table 1. Across all participants, there were significant differences in age, race, BMI, CVD events, hypertension, and LCD score between those with and without CKD (all *P* < 0.05). Table 1 indicates that the participants’ mean age was 45.56 years (45.56 ± 11.95), and 58.3% of them were non-Hispanic white. Specifically, individuals with CKD may be older (49.16 ± 12.56 vs. 44.93 ± 11.74, *P* = 0.012), more likely to be obese (70.9%), have higher rates of hypertension (691%); although, the majority do not have a history of CVD (84.7%). CKD patients possessed higher LCD scores (13.95 ± 6.83 vs. 11.9 ± 7.66, *P* = 0.049).

**Table 1:** Characteristics of NHANES participants between 2009 and 2018

Characters	Total	Non-CKD	CKD	*P*
**Overall**	701	571 (85.2)	130 (14.8)	
**Age**	45.56 (11.95)	44.93 (11.74)	49.16 (12.56)	0.012
**Race**				0.048
Mexican American	140 (13.5)	113 (12.8)	27 (17.3)	
Other Hispanic	73 (6.9)	57 (6.4)	16 (9.9)	
Non-Hispanic White	253 (58.3)	211 (62.0)	42 (36.9)	
Non-Hispanic Black	131 (9.5)	98 (8.7)	33 (14.0)	
Other race	104 (11.9)	92 (10.1)	12 (21.9)	
**BMI (kg/m** ^2^ **)**				0.034
<25	127 (20.5)	107 (21.2)	20 (16.7)	
25–30	186 (22.9)	161 (24.8)	25 (12.4)	
≥30	388 (56.6)	303 (54.1)	85 (70.9)	
**Smoking**				0.788
Never smoking	444 (63.8)	369 (63.4)	75 (66.2)	
Former smoking	131 (21.2)	102 (21.8)	29 (17.9)	
Now Smoking	126 (15.0)	100 (14.8)	26 (15.9)	
**Alcohol drinking**				0.765
No	236 (27.8)	195 (27.5)	41 (29.7)	
Yes	465 (72.2)	376 (72.5)	89 (70.3)	
**CVD events**				0.003
No	635 (92.4)	531 (93.7)	104 (84.7)	
Yes	66 (7.6)	40 (6.3)	26 (15.3)	
**Hypertension**				0.004
No	336 (51.0)	297 (54.5)	39 (30.9)	
Yes	365 (49.0)	274 (45.5)	91 (69.1)	
**Family history of diabetes**				0.393
No	232 (33.2)	201 (31.9)	31 (40.0)	
Yes	469 (66.9)	370 (68.1)	99 (60.0)	
**PIR**				0.424
Low	261 (26.2)	202 (24.9)	59 (33.6)	
Medium	248 (34.2)	202 (34.2)	46 (34.4)	
High	192 (39.6)	167 (41.0)	25 (31.9)	
**LCD score**	12.23 (7.6)	11.94 (7.66)	13.95 (6.83)	0.049
Total fat (g)	71.48 (31.19)	70.89 (30.56)	74.90 (34.52)	0.353
Protein (g)	73.44 (26.87)	73.33 (27.12)	74.12 (25.49)	0.776
Carbohydrate (g)	215.32 (86.4)	218.08 (88.73)	199.38 (69.80)	0.082
Energy (kcal)	1806.33 (625.86)	1812.45 (630.80)	1770.98 (597.86)	0.624

Note: Categorical variables are expressed as *n* (%) and continuous variables are expressed as mean (sd); *n* is unweighted and *n* (%), mean and standard deviation are weight-adjusted.

### Stratified analysis

A weighted logistic regression model was created to examine the link between LCD scores and CKD. Stratified analysis (Table 2) indicated that LCD scores were positively linked to risk of CKD in the population with now smoking (odds ratio [OR] = 1.14, 95% confidence interval [CI]: 1.05–1.25, *P* = 0.001), alcohol drinking (OR = 1.04, 95% CI: 1.00–1.09, *P* = 0.040), hypertension (OR = 1.05, 95% CI: 1.00–1.10, *P* = 0.041), and medium PIR (OR = 1.07, 95% CI: 1.01–1.13, *P* = 0.024). Low-carbohydrate-diet scores and CKD were strongly influenced by race, BMI, and smoking, according to the findings of the interaction test between confounders and LCD scores (*P* for interaction < 0.05).

**Table 2:** Relationship between LCD score and CKD in categorical variables in GDM

Participants	OR	95% CI	*P*	*P* for interaction
**Race**				<0.001
Mexican American	1.00	0.96–1.04	>0.900	
Other Hispanic	1.07	0.94–1.22	0.300	
Non-Hispanic White	1.02	0.97–1.07	0.500	
Non-Hispanic Black	1.08	0.99–1.17	0.057	
Other race	1.17	0.97–1.43	0.069	
**BMI (kg/m** ^2^ **)**				0.027
<25	1.09	0.99–1.21	0.062	
25–30	0.97	0.91–1.03	0.300	
≥30	1.04	0.99–1.09	0.110	
**Smoking**				0.007
Never smoking	1.02	0.96–1.07	0.600	
Former smoking	1.04	0.96–1.13	0.300	
Now Smoking	1.14	1.05–1.25	0.001	
**Alcohol drinking**				0.338
No	1.01	0.94–1.09	0.800	
Yes	1.04	1.00–1.09	0.040	
**CVD events**				0.633
No	1.04	1.00–1.08	0.056	
Yes	1.04	0.92–1.16	0.500	
**Hypertension**				0.152
No	1.00	0.95–1.05	>0.900	
Yes	1.05	1.00–1.10	0.041	
**PIR**				0.127
Low	1.05	01.00–1.10	0.062	
Medium	1.07	1.01–1.13	0.024	
High	1.03	0.95–1.11	0.500	
**Family history of diabetes**				0.802
No	1.04	0.97–1.12	0.300	
Yes	1.03	1.00–1.07	0.086	

Note: Interaction term *P*-values adjusted for age, race, BMI, smoking, alcohol drinking, CVD events, hypertension, family history of diabetes, and PIR.

### Sub-group analysis

We next stratified the LCD scores using weighted tertiles and constructed weighted logistic regression models for LCD scores and CKD to explore the association between them. Table 3 demonstrates that, in the unstratified population, the LCD score was substantially and positively linked with CKD after adjusting for various confounding factors in both model I (OR: 1.06, 95% CI: 1.02–1.09, *P* < 0.01) and model II (OR: 1.06, 95% CI: 1.02–1.09, *P* < 0.01). Additional tertile stratification of LCD scores revealed that, in comparison to LCD scores in the first quartile (Q1), the second and third quartiles (Q2, Q3) in all three models significantly increased the risk of CKD in women with a history of GDM (OR > 1, *P* < 0.05).

**Table 3:** Associations between LCD score and odds ratios (95% confidence intervals) for CKD in GDM

Participants	OR (95% CI)		
	Crude	Model I	Model II
**All participants**	1.04 (1.00–1.07)	1.06 (1.02–1.09) **	1.06 (1.02–1.09) **
**LCD** score			
Q1 (≤8)	Ref.	Ref.	Ref.
Q2 (8–16)	1.37 (0.70–2.72)	1.62 (0.74–3.56)	1.58 (0.73–3.40)
Q3 (>16)	2.37 (1.15–4.86) *	3.34 (1.65–6.75) **	3.40 (1.73–6.70) ***
***P* for trend**	**0.022**	**<0.001**	**<0.001**

Note: Crude unadjusted; model I adjusted for age, race, BMI, smoking, alcohol drinking, and PIR; model II adjusted for age, race, BMI, smoking, alcohol drinking, PIR, hypertension, CVD events, and family history of diabetes.

* *P*-value < 0.05,

** *P*-value < 0.01,

*** *P*-value < 0.001.

Sub-group analyses (Table 4) based on alcohol drinking and smoking yielded substantial positive associations between LCD scores and CKD risk among current alcohol users and smokers. These associations persisted in all models even after adjusting for confounders (all OR > 1, *P* < 0.05).

**Table 4:** Relationship between LCD score and CKD by smoking (95% CI)

Participants	OR (95% CI)		
	Crude	Model I	Model II
**Smoking**			
Never smoking	1.02 (0.96–1.07)	1.02 (0.97–1.07)	1.02 (0.98–1.07)
Former smoking	1.04 (0.96–1.13)	1.04 (0.95–1.15)	1.04 (0.95–1.15)
Now Smoking	1.14 (1.05–1.25) **	1.18 (1.07–1.29) ***	1.20 (1.07–1.34) ***
**Alcohol drinking**			
No	1.01 (0.94–1.09)	1.00 (0.93–1.08)	1.00 (0.92–1.07)
Yes	1.04 (1.00–1.09) *	1.07 (1.03–1.11) ***	1.07 (1.03–1.11) ***

Note: Crude unadjusted; model I adjusted for age, race, BMI, smoking, alcohol drinking, and PIR; model II adjusted for age, race, BMI, smoking, alcohol drinking, hypertension, family history of diabetes, PIR, and CVD events.

* *P*-value < 0.05,

** *P*-value < 0.01,

*** *P*-value < 0.001.

### Non-linear association between LCD score and CKD risk

**Fig. 2:**
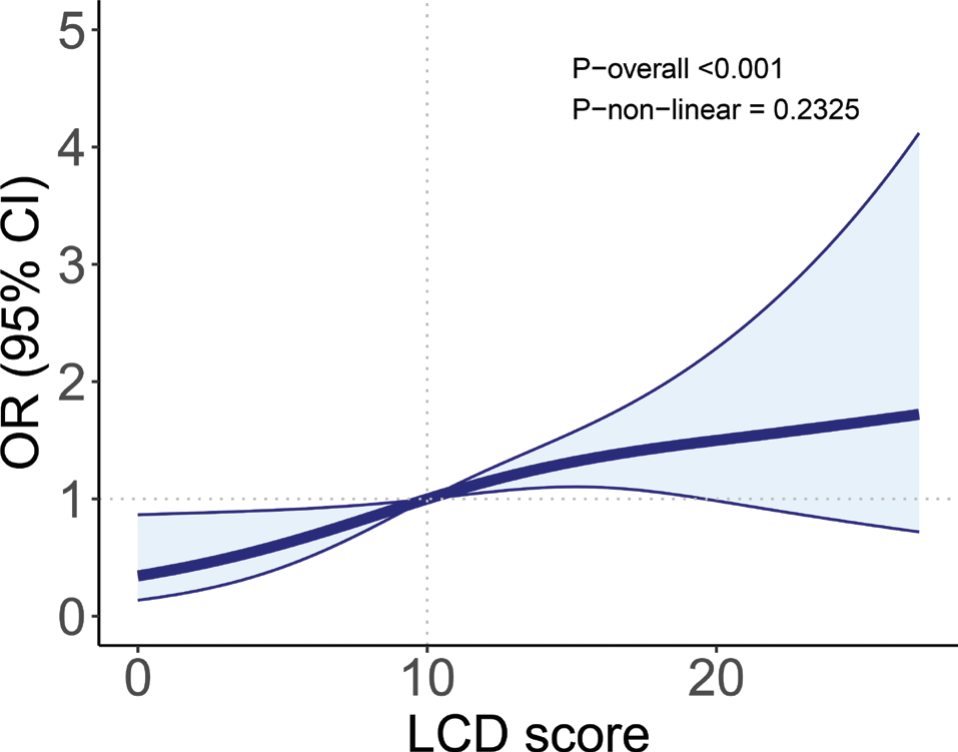
The OR of LCD score and CKD adjusted by covariates in GDM, NHANES 2009–2018. Note: RCS line is adjusted for age, race, BMI, smoking, alcohol, hypertension, family history of diabetes, PIR, CVD events. The OR is represented by the blue line and the shaded part represents the 95% CI.

Figure 2 displays RCS analysis of association between LCD score and CKD risk. A significant overall trend between LCD score and CKD risk was seen (*P* < 0.001), with the possibility of a linear relationship detected in the model adjusting for all confounding factors (*P*-non-linear = 0.2325). Restricted cubic splines curves showed a risk factor when LCD > 10 and OR > 1, and a protective factor when LCD <10 and OR < 1.

## Discussion

In this study, women with a history of GDM showed a substantial positive correlation between their LCD score and their chance of developing CKD. Adverse behaviours like drinking and smoking can be used as an explanation for these linked components. A study of the RCS curve revealed a linearly positive correlation between the probability of developing CKD and the LCD score, with a threshold inflection point at LCD score = 10.

According to earlier research, women with a history of GDM are more likely to show early indicators of impaired renal function after delivery, such as elevated urine albumin excretion (25) or increased glomerular filtration rate (26). There is now growing evidence that GDM is linked to an increased risk of developing CKD (25, 27, 28). The results of our study indicate that there may be a connecting factor impacting the relationship between LCD and the risk of developing CKD in women with a history of GDM, since there was a substantial positive association discovered between the two variables.

In a healthy diet, carbohydrates, lipids, and proteins are highly desirable since they are regarded as vital elements for human health (29). By modifying lipid metabolism in gastrointestinal illnesses, liver disease, and CKD, nutrition-based therapies can achieve prognosis improvement (30,31,32). Low carbohydrate, high fat, and protein diets are frequent patterns for weight reduction and glycaemic management in women with GDM. These dietary patterns are directly linked to the preservation of the body’s physical health (5). In comparison to a conventional diet, a greater LCD will have a larger proportion of protein. Higher protein consumption might raise the risk of impaired renal function and glomerular filtration rate overload in people, which can accelerate the course of CKD (33,34,35). A high consumption of animal protein was linked to an elevated risk of incident CKD in prospective research on dietary meat intake and the risk of developing CKD (36). Furthermore, a prolonged high-fat diet will cause an imbalance between the body’s intake and use of energy, which will ultimately result in issues related to lipid metabolism, raising the risk of CKD (37). Our work offers important insights for future more scientific dietary treatment of GDM patients, as it appears to be the first to examine the association between LCD and the risk of CKD in this population.

This study revealed that alcohol and smoking may have an impact on the correlation between LCD score and CKD risk. It is often recognised that consuming alcohol and smoking are frequent risk factors for human health, having the ability to both cause and exacerbate a wide range of disorders. Alcohol intake and odor boost the body’s desire for carbs, lipids, and proteins, which increases food intake (38, 39). Alcohol increases hunger and delays satiety. Through the activation of brain neurons that generate neuropeptide Y and hormones associated to function, alcohol can regulate appetite by inducing hunger and stimulating the generation of eating behaviour (40, 41). Besides, we think that the body’s energy demands shift to the levels of protein and fat as a result of the LCD mode’s reduction in carbohydrate consumption, particularly in the high-protein and high-fat LCD from animal sources. The animal-based high LCD score group consumed more alcohol, according to a prior study conducted on the emergence of metabolic disorders with LCD (42).

Smoking is another risk factor that influences the LCD pattern, much like alcohol consumption. Smokers could choose less healthily when it comes to food than non-smokers and ex-smokers. According to research on smokers’ food and drink cravings, high-fat meals, coffee, and alcoholic beverages can all cause desires for smoking, and smokers consume much less fruit and dairy each day than non-smokers (43). According to a cross-sectional study comparing the dietary quality of current and past smokers with that of never smokers, former smokers often consume more healthful foods (such fruits, vegetables, whole grains, proteins, and fatty acids) than do current smokers (44). In a similar vein, low food quality was linked to current smoking status in a prior study on the connection between dietary energy density and smoking status among adult Americans (45). The risk of CKD is strongly correlated with the use of high-fat foods and diabetes (46). Specifically, a diet heavy in animal fat and protein, like the LCD diet, increases the risk of developing and dying from CKD (47, 48). Thus, we hypothesise that when on an LCD diet, women with a history of GDM may overindulge in high-fat foods to fulfil their want for tobacco, raising their chance of developing CKD (49).

We admit that there are certain restrictions. Firstly, the NHANES database served as the basis for this retrospective study. A significant number of patients were excluded from the final analysis due to a lack of data availability, which might have an impact on the discrepancy between the observed and real incidence of CKD. Secondly, there is no indication of a causal relationship between the two by this link. There could still be confounding variables that affect the results even if we tried to control them via multivariate adjustments and sub-group analysis. Thirdly, American women made up the majority of the research population. As such, extrapolating our study’s findings to other areas and demographics might prove difficult.

## Conclusion

In conclusion, the risk of CKD is highly linked with LCD score among women who have a history of GDM. It may not be suitable to blindly follow a low-carb diet when on an LCD pattern in people with a history of GDM. Further research is required to design a more realistic dietary management plan that will lower the risk of eventual CKD.

## Declaration

### Availability of data and materials

All the data within this manuscript are available from the corresponding author at reasonable request.

## References

[1] Kautzky-Willer A, Winhofer Y, Kiss H, Falcone V, Berger A, Lechleitner M, et al. [Gestational diabetes mellitus (Update 2023)]. Wien Klin Wochenschr 2023; 135 (Suppl 1): 115–28. doi: 10.1007/s00508-023-02181-9 37101032 PMC10132924

[2] Pande B, Verma HK, Bhaskar L. Tailored nutritional interventions: a precision approach to managing gestational diabetes mellitus. World J Diabetes 2024; 15(5): 1045–7. doi: 10.4239/wjd.v15.i5.1045 38766438 PMC11099369

[3] Mustafa S, Harding J, Wall C, Crowther C. Sociodemographic factors associated with adherence to dietary guidelines in women with gestational diabetes: a cohort study. Nutrients 2021; 13(6): 1884. doi: 10.3390/nu13061884 34072685 PMC8228016

[4] Tsakiridis I, Giouleka S, Mamopoulos A, Kourtis A, Athanasiadis A, Filopoulou D, et al. Diagnosis and management of gestational diabetes mellitus: an overview of national and international guidelines. Obstet Gynecol Surv 2021; 76(6): 367–81. doi: 10.1097/ogx.0000000000000899 34192341

[5] Oh R, Gilani B, Uppaluri KR. Low-carbohydrate diet. StatPearls Publishing LLC.; Tampa; 2024. 30725769

[6] Ammirati AL. Chronic kidney disease. Rev Assoc Med Bras (1992) 2020; 66(Suppl 1): s03–9. doi: 10.1590/1806-9282.66.S1.3 31939529

[7] Deng Y, Li N, Wu Y, Wang M, Yang S, Zheng Y, et al. Global, regional, and national burden of diabetes-related chronic kidney disease from 1990 to 2019. Front Endocrinol (Lausanne) 2021; 12: 672350. doi: 10.3389/fendo.2021.672350 34276558 PMC8281340

[8] Vondenhoff S, Schunk SJ, Noels H. Increased cardiovascular risk in patients with chronic kidney disease. Herz 2024; 49(2): 95–104. doi: 10.1007/s00059-024-05235-4 38416185 PMC10917854

[9] Noels H, Jankowski J. Increased risk of cardiovascular complications in chronic kidney disease: introduction to a compendium. Circ Res 2023; 132(8): 899–901. doi: 10.1161/circresaha.123.322806 37053281

[10] Chen HY, Lu FH, Chang CJ, Wang RS, Yang YC, Chang YF, et al. Metabolic abnormalities, but not obesity per se, associated with chronic kidney disease in a Taiwanese population. Nutr Metab Cardiovasc Dis 2020; 30(3): 418–25. doi: 10.1016/j.numecd.2019.09.029 31744713

[11] Papadaki A, Nolen-Doerr E, Mantzoros CS. The effect of the mediterranean diet on metabolic health: a systematic review and meta-analysis of controlled trials in adults. Nutrients 2020; 12(11): 3342. doi: 10.3390/nu12113342 33143083 PMC7692768

[12] Zhang N, Cheng Y, Luo R, Chang D, Liu T, Wang Z, et al. Low-carbohydrate-diet score and mortality in adults with and without chronic kidney disease: results from the third national health and nutrition examination survey. J Ren Nutr 2022; 32(3): 301–11. doi: 10.1053/j.jrn.2021.05.004 34972598

[13] Li L, Ji J, Li Y, Huang YJ, Moon JY, Kim RS. Gestational diabetes, subsequent type 2 diabetes, and food security status: national health and nutrition examination survey, 2007–2018. Prev Chronic Dis 2022; 19: E42. doi: 10.5888/pcd19.220052 35834736 PMC9336195

[14] Halton TL, Willett WC, Liu S, Manson JE, Albert CM, Rexrode K, et al. Low-carbohydrate-diet score and the risk of coronary heart disease in women. N Engl J Med 2006; 355(19): 1991–2002. doi: 10.1056/NEJMoa055317 17093250

[15] Wang H, Lv Y, Ti G, Ren G. Association of low-carbohydrate-diet score and cognitive performance in older adults: National Health and Nutrition Examination Survey (NHANES). BMC Geriatr 2022; 22(1): 983. doi: 10.1186/s12877-022-03607-1 36539697 PMC9764565

[16] Deng Y, Zhao Q, Gong R. Association between metabolic associated fatty liver disease and chronic kidney disease: a cross-sectional study from NHANES 2017–2018. Diabetes Metab Syndr Obes 2021; 14: 1751–61. doi: 10.2147/dmso.S292926 33911888 PMC8075735

[17] Levey AS, Stevens LA. Estimating GFR using the CKD Epidemiology Collaboration (CKD-EPI) creatinine equation: more accurate GFR estimates, lower CKD prevalence estimates, and better risk predictions. Am J Kidney Dis 2010; 55(4): 622–7. doi: 10.1053/j.ajkd.2010.02.337 20338463 PMC2846308

[18] Guo W, Song Y, Sun Y, Du H, Cai Y, You Q, et al. Systemic immune-inflammation index is associated with diabetic kidney disease in Type 2 diabetes mellitus patients: evidence from NHANES 2011–2018. Front Endocrinol (Lausanne) 2022; 13: 1071465. doi: 10.3389/fendo.2022.1071465 36561561 PMC9763451

[19] Zhang Y, Liu W, Zhang W, Cheng R, Tan A, Shen S, et al. Association between blood lead levels and hyperlipidemiais: results from the NHANES (1999–2018). Front Public Health 2022; 10: 981749. doi: 10.3389/fpubh.2022.981749 36159291 PMC9500574

[20] Li X, Zhao Y, Zhang D, Kuang L, Huang H, Chen W, et al. Development of an interpretable machine learning model associated with heavy metals’ exposure to identify coronary heart disease among US adults via SHAP: findings of the US NHANES from 2003 to 2018. Chemosphere 2023; 311(Pt 1): 137039. doi: 10.1016/j.chemosphere.2022.137039 36342026

[21] Li C, Shang S. Relationship between sleep and hypertension: findings from the NHANES (2007–2014). Int J Environ Res Public Health 2021; 18(15): 7867. doi: 10.3390/ijerph18157867 34360157 PMC8345503

[22] Miao H, Liu Y, Tsai TC, Schwartz J, Ji JS. Association between blood lead level and uncontrolled hypertension in the US population (NHANES 1999–2016). J Am Heart Assoc 2020; 9(13): e015533. doi: 10.1161/jaha.119.015533 32573312 PMC7670543

[23] Moonesinghe R, Beckles GLA, Liu T, Khoury MJ. The contribution of family history to the burden of diagnosed diabetes, undiagnosed diabetes, and prediabetes in the United States: analysis of the National Health and Nutrition Examination Survey, 2009–2014. Genet Med 2018; 20(10): 1159–66. doi: 10.1038/gim.2017.238 29369292 PMC6060023

[24] Liao S, Zhang J, Shi S, Gong D, Lu X, Cheang I, et al. Association of aldehyde exposure with cardiovascular disease. Ecotoxicol Environ Saf 2020; 206: 111385. doi: 10.1016/j.ecoenv.2020.111385 33010595

[25] Dehmer EW, Phadnis MA, Gunderson EP, Lewis CE, Bibbins-Domingo K, Engel SM, et al. Association between gestational diabetes and incident maternal CKD: the Coronary Artery Risk Development in Young Adults (CARDIA) study. Am J Kidney Dis 2018; 71(1): 112–22. doi: 10.1053/j.ajkd.2017.08.015 29128412 PMC5742081

[26] Rawal S, Olsen SF, Grunnet LG, Ma RC, Hinkle SN, Granström C, et al. Gestational diabetes mellitus and renal function: a prospective study with 9- to 16-year follow-up after pregnancy. Diabetes Care 2018; 41(7): 1378–84. doi: 10.2337/dc17-2629 29728364 PMC6014536

[27] Christensen MH, Bistrup C, Rubin KH, Nohr EA, Vinter CA, Andersen MS, et al. Kidney disease in women with previous gestational diabetes mellitus: a nationwide register-based cohort study. Diabetes Care 2024; 47(3): 401–8. doi: 10.2337/dc23-1092 38100751

[28] Crump C, Sundquist J, Sundquist K. Adverse pregnancy outcomes and long-term risk of chronic kidney disease in women: national cohort and co-sibling study. Am J Obstet Gynecol 2024; 230(5): 563.e561–3.e520. doi: 10.1016/j.ajog.2023.10.008 PMC11006822 37827269

[29] Zohoori FV. Chapter 1: nutrition and diet. Monogr Oral Sci 2020; 28: 1–13. doi: 10.1159/000455365 31940634

[30] Gluba-Brzozka A, Franczyk B, Rysz J. Cholesterol disturbances and the role of proper nutrition in CKD patients. Nutrients 2019; 11(11): 2820. doi: 10.3390/nu11112820 31752189 PMC6893650

[31] Ukleja A. Nutritional management of gastrointestinal diseases. Gastroenterol Clin North Am 2018; 47(1): xv–xx. doi: 10.1016/j.gtc.2017.12.001 29413028

[32] Bischoff SC, Bernal W, Dasarathy S, Merli M, Plank LD, Schütz T, et al. [ESPEN Practical Guideline: clinical nutrition in liver disease]. Nutr Hosp 2022; 39(2): 434–72. doi: 10.20960/nh.03856 35014850

[33] Oba R, Kanzaki G, Sasaki T, Okabayashi Y, Haruhara K, Koike K, et al. Dietary protein intake and single-nephron glomerular filtration rate. Nutrients 2020; 12(9): 2549. doi: 10.3390/nu12092549 32842498 PMC7551595

[34] Ko GJ, Rhee CM, Kalantar-Zadeh K, Joshi S. The effects of high-protein diets on kidney health and longevity. J Am Soc Nephrol 2020; 31(8): 1667–79. doi: 10.1681/asn.2020010028 32669325 PMC7460905

[35] Kramer H. Diet and chronic kidney disease. Adv Nutr 2019; 10 (Suppl_4): S367–79. doi: 10.1093/advances/nmz011 31728497 PMC6855949

[36] Mirmiran P, Yuzbashian E, Aghayan M, Mahdavi M, Asghari G, Azizi F. A prospective study of dietary meat intake and risk of incident chronic kidney disease. J Ren Nutr 2020; 30(2): 111–8. doi: 10.1053/j.jrn.2019.06.008 31422013

[37] Pei K, Gui T, Li C, Zhang Q, Feng H, Li Y, et al. Recent progress on lipid intake and chronic kidney disease. Biomed Res Int 2020; 2020: 3680397. doi: 10.1155/2020/3680397 32382547 PMC7196967

[38] Bae D, Wróbel A, Kaelin I, Pestoni G, Rohrmann S, Sych J. Investigation of alcohol-drinking levels in the Swiss population: differences in diet and associations with sociodemographic, lifestyle and anthropometric factors. Nutrients 2022; 14(12): 2494. doi: 10.3390/nu14122494 35745224 PMC9230148

[39] Joseph PV, Zhou Y, Brooks B, McDuffie C, Agarwal K, Chao AM. Relationships among alcohol drinking patterns, macronutrient composition, and caloric intake: national health and nutrition examination survey 2017–2018. Alcohol Alcohol 2022; 57(5): 559–65. doi: 10.1093/alcalc/agac009 35284941 PMC9465521

[40] Chen YW, Barson JR, Chen A, Hoebel BG, Leibowitz SF. Hypothalamic peptides controlling alcohol intake: differential effects on microstructure of drinking bouts. Alcohol 2014; 48(7): 657–64. doi: 10.1016/j.alcohol.2014.08.005 25241055 PMC4250297

[41] Carvajal F, Alcaraz-Iborra M, Lerma-Cabrera JM, Valor LM, de la Fuente L, Sanchez-Amate Mdel C, et al. Orexin receptor 1 signaling contributes to ethanol binge-like drinking: pharmacological and molecular evidence. Behav Brain Res 2015; 287: 230–7. doi: 10.1016/j.bbr.2015.03.046 25827928

[42] Nakamura Y, Ueshima H, Okuda N, Miura K, Kita Y, Miyagawa N, et al. Relationship of three different types of low-carbohydrate diet to cardiometabolic risk factors in a Japanese population: the INTERMAP/INTERLIPID Study. Eur J Nutr 2016; 55(4): 1515–24. doi: 10.1007/s00394-015-0969-z 26119583 PMC6697100

[43] Miyoshi K, Kimura Y, Nakahata M, Miyawaki T. Foods and beverages associated with smoking craving in heated tobacco product and cigarette smokers: a cross-sectional study. Tob Induc Dis 2024; 22: 175623. doi: 10.18332/tid/175623 PMC10767721 38188940

[44] Luo T, Tseng TS. Diet quality as assessed by the healthy eating index-2020 among different smoking status: an analysis of national health and nutrition examination survey (NHANES) data from 2005 to 2018. BMC Public Health 2024; 24(1): 1212. doi: 10.1186/s12889-024-18630-7 38693488 PMC11064397

[45] MacLean RR, Cowan A, Vernarelli JA. More to gain: dietary energy density is related to smoking status in US adults. BMC Public Health 2018; 18(1): 365. doi: 10.1186/s12889-018-5248-5 29614996 PMC5883399

[46] Hidayangsih PS, Tjandrarini DH, Sukoco NEW, Sitorus N, Dharmayanti I, Ahmadi F. Chronic kidney disease in Indonesia: evidence from a national health survey. Osong Public Health Res Perspect 2023; 14(1): 23–30. doi: 10.24171/j.phrp.2022.0290 36944342 PMC10211444

[47] Farhadnejad H, Asghari G, Emamat H, Mirmiran P, Azizi F. Low-carbohydrate high-protein diet is associated with increased risk of incident chronic kidney diseases among Tehranian adults. J Ren Nutr 2019; 29(4): 343–9. doi: 10.1053/j.jrn.2018.10.007 30579675

[48] Joshi S, Kalantar-Zadeh K, Chauveau P, Carrero JJ. Risks and benefits of different dietary patterns in CKD. Am J Kidney Dis 2023; 81(3): 352–60. doi: 10.1053/j.ajkd.2022.08.013 36682903

[49] McClernon FJ, Westman EC, Rose JE, Lutz AM. The effects of foods, beverages, and other factors on cigarette palatability. Nicotine Tob Res 2007; 9(4): 505–10. doi: 10.1080/14622200701243177 17454706

